# Nanolayer encapsulation of insulin-chitosan complexes improves efficiency of oral insulin delivery

**DOI:** 10.2147/IJN.S59075

**Published:** 2014-05-02

**Authors:** Lei Song, Zheng-liang Zhi, John C Pickup

**Affiliations:** Diabetes Research Group, King’s College London School of Medicine, Guy’s Hospital, London, United Kingdom

**Keywords:** oral insulin, diabetes mellitus, insulin-chitosan complexes, multilayer nanoencapsulation, polyethylene glycol, chitosan, heparin

## Abstract

Current oral insulin formulations reported in the literature are often associated with an unpredictable burst release of insulin in the intestine, which may increase the risk for problematic hypoglycemia. The aim of the study was to develop a solution based on a nanolayer encapsulation of insulin-chitosan complexes to afford sustained release after oral administration. Chitosan/heparin multilayer coatings were deposited onto insulin-chitosan microparticulate cores in the presence of poly(ethylene) glycol (PEG) in the precipitating and coating solutions. The addition of PEG improved insulin loading and minimized an undesirable loss of the protein resulting from redissolution. Nanolayer encapsulation and the formation of complexes enabled a superior loading capacity of insulin (>90%), as well as enhanced stability and 74% decreased solubility at acid pH in vitro, compared with nonencapsulated insulin. The capsulated insulin administered by oral gavage lowered fasting blood glucose levels by up to 50% in a sustained and dose-dependent manner and reduced postprandial glycemia in streptozotocin-induced diabetic mice without causing hypoglycemia. Nanolayer encapsulation reduced the possibility of rapid and erratic falls of blood glucose levels in animals. This technique represents a promising strategy to promote the intestinal absorption efficiency and release behavior of the hormone, potentially enabling an efficient and safe route for oral insulin delivery of insulin in diabetes management.

## Introduction

Insulin is the major protein hormone synthesized by the β cells of the pancreatic islets of Langerhans. It is essential for the treatment of type 1 diabetes and is often needed for optimal control of type 2 diabetes mellitus. It is usually administered to diabetic patients by subcutaneous injection. However, poor glycemic control in many people with insulin-treated diabetes is in large part a result of the restrictions of subcutaneous insulin delivery; in particular, the need to inject insulin frequently leads to discomfort, inconvenience, an impaired lifestyle, and poor compliance.^1^ Many patients with type 2 diabetes who are poorly controlled on oral hypoglycemic agents also resist or delay a switch to insulin because of fear of injection.

An oral delivery route of insulin would be the most convenient, comfortable, and patient-preferable means of administering the hormone. In addition, oral insulin, by being absorbed into the hepatic portal circulation, mimics the physiological route of insulin delivery to the liver, thereby reducing the systemic hyperinsulinemia associated with the subcutaneous injection that delivers insulin to the peripheral circulation, and with the potential to minimize risk for hypoglycemia and improve metabolic control.^2^

The main barriers to the intestinal absorption of insulin and a clinically usable oral insulin formulation are the low permeability of proteins across the intestinal wall coupled with high susceptibility to acid denaturation in the stomach and enzymatic degradation throughout the gut. Various strategies for improving gastrointestinal uptake of insulin have been explored, including using carrier systems in which insulin was entrapped in constructs such as nanospheres or nanoparticles,^3^–^9^ microparticles,^10^,^11^ or liposomes.^12^,^13^ These systems are intended to have the dual role of shielding insulin from the proteolytic/denaturing processes in the upper gastrointestinal tract and enhancing transmucosal uptake of the hormone from various regions of the small intestine. Problems associated with micellar/liposomal or similar carriers to date^14^ include poor loading efficacy of insulin into the capsules and erratic control of the release rate of the protein from the capsules. Thus, significant hurdles remain in both the engineering of micro- and nanosystems as insulin carriers and the achievement of high-efficiency, predictable oral insulin delivery at low cost.

Chitosan, a cationic naturally occurring polysaccharide composed of β(1-4)-linked D-glucosamine and *N*-acetyl-D-glucosamine, has been extensively exploited for the preparation of micro/nanoparticles as potential carriers because it enhances the bioadhesive properties and the transmucosal absorption of insulin.^15^,^16^ Chitosan spontaneously associates with negatively charged insulin in solution to form insoluble complexes. The mucoadhesive property of chitosan extends contact time and is coupled with a transient opening of tight junctions between the epithelial cells, thus promoting paracellular permeability. However, insulin-entrapped chitosan particles have poor colloidal stability and are pH-responsive, readily dissociating and dissolving in the acidic gastric conditions.^17^ Thus, although the therapeutic effect is fast and intense after oral administration of insulin-chitosan nanoparticles, the oral pharmacological activity is highly variable.^2^ Another consequence of unpredictable burst release and action is that it may induce a rapid or erratic fall in circulating glucose level, leading to problematic hypoglycemia when used clinically.

Here we present a novel application of ultrathin nanofilm layer-by-layer (LBL) encapsulation technology (hereafter called nanoencapsulation) in which insulin-chitosan microparticles were formulated with very high content (>90% by weight) of the bioactive protein. These microparticles were surface coated with a multilayer but nanothickness shell comprising chitosan and heparin to give an acid-stable and sustained release formulation; this formulation was tested as a potential oral insulin delivery system in a diabetic mouse model.

## Materials and methods

### Materials

Recombinant human insulin solution (10 mg/mL in 25 mM hydroxyethyl piperazineethanesulfonic acid (HEPES) at pH 8.2; activity, 27 units/mg), fluorescein-5-isothiocyanate (FITC), dimethylsulfoxide, streptozotocin, and chitosan (low molecular weight, 50–190 kDa, 75% deacetylation) were obtained from Sigma-Aldrich (Poole, UK). Heparin sodium salt was provided by AppliChem (Darmstadt, Germany). Poly(ethylene) glycol (PEG) 6000 was purchased from BDH Laboratory Supplies (Poole, UK).

### Synthesis of chitosan-FITC

FITC was dissolved in dimethylsulfoxide (1 mg/mL). Chitosan (200 μL or 2 mg), taken from a 1% weight/volume (W/V) stock solution (dissolved in 1% acetic acid), was diluted to 1 mL with 50 mM HEPES at pH 8.1. FITC solution (0.1 mL) was then added to the chitosan solution and mixed overnight at 4°C. To remove excess dye, the solution was dialyzed against water using Slide-A-Lyzer 3.5k dialysis cassettes (Pierce, Thermo Fisher Scientific Inc., Rockford, IL, USA) for 16 hours, and then 50 mM HEPES buffer for 4 hours. This solution, which was frozen at −20°C during storage, was used for encapsulating the insulin-containing microparticles as a fluorescence-labeled polycation layer.

### Preparation of insulin microparticles by precipitation in PEG

Insulin microparticles were made by adding 50 μL insulin stock solution (10 mg/mL) to 50 μL 20% PEG 6000 (in 0.05 M phosphate buffer at pH 5.5) with stirring at 4°C for 1 hour, followed by centrifugation (5,600× *g* for 3 minutes; MSE Micro Centaur microcentrifuge, MSE Ltd, London, UK) to collect the solids.

The precipitation yield of insulin was calculated by measuring the decrease in ultraviolet (UV) absorbance at 280 nm of the supernatant after centrifugation (quantified for the protein concentration using standard insulin solutions), using a quartz cuvette measured in an Ultrospec 2100 Pro UV/visible spectrophotometer (GE Healthcare UK Ltd, Little Chalfont, UK). The measured UV absorbance must be a result of the dissolved insulin in solution, as none of the chemical groups in chitosan or heparin or PEG has a substantial absorption dipole moment near 280 nm. This was confirmed in our experiments (data not shown).

### Preparation of insulin-chitosan microparticles by complexation-precipitation in PEG

Insulin-chitosan microparticles were prepared by mixing 50 μL insulin solution (10 mg/mL) with 50 μL of 1 mg/mL chitosan in 20% PEG 6000 (in 0.05 M phosphate buffer at pH 5.5) with stirring at 4°C for 1 hour, followed by collection of the solids by centrifugation (5,600× *g*, 3 minutes). The precipitation yield of insulin was determined by measuring the supernatant UV absorbance at 280 nm.

### Nanocoating of insulin and insulin-chitosan microparticles

**Figure 1:**
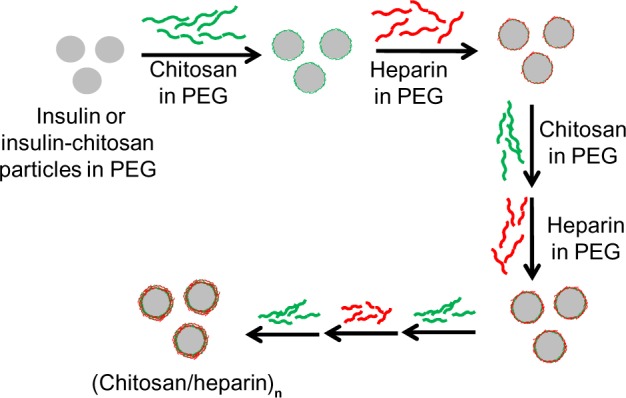
Scheme of nanocoating of insulin or insulin-chitosan microparticles in the nanoengineered polysaccharide capsules. **Notes:** The insulin microparticles were formed in 10% PEG 6000, and alternating layers of chitosan and heparin were then applied in the presence of 10% PEG, thereby forming multilayers encapsulating insulin or insulin-chitosan microparticles. **Abbreviation:** PEG, poly(ethylene) glycol.

The nanocoating procedure was carried out as shown schematically in Figure 1. Insulin or insulin-chitosan microparticles were encapsulated using an LBL deposition scheme. Briefly, the microparticles were first coated by the addition of 50 μL 1 mg/mL chitosan solution prepared in 10% PEG 6000 (in 0.05 M phosphate saline buffer at pH 5.5) at 4°C and then collected by centrifugation (5,600× *g*, 3 minutes). The recovered microparticles were rinsed twice by the addition of 50 μL 10% PEG 6000 solution and then coated by the addition of 50 μL 1 mg/mL heparin solution prepared in 10% PEG 6000, which was again followed by rinsing steps. By repeating the procedure, microcapsules with multilayers were obtained. The scheme for the microcapsule was insulin or insulin-chitosan:(chitosan/heparin)_n_ chitosan, with the outermost layer of chitosan maintaining the positive charge or mucoadhesive property that may play an important role in enhancing the gastrointestinal uptake of insulin.

The insulin percentage loss during particle rinsing and sequential deposition of the chitosan/heparin layers was measured by UV absorbance (at 280 nm, the aromatic absorbance peak) of supernatants collected and then calibrated, using standard insulin solutions.

### pH-dependent dissolution and morphology of the nanocoated microparticles

In a typical experiment, insulin and insulin-chitosan microparticles were coated with seven layers of chitosan/heparin, with the first layer being FITC-labeled chitosan. A small drop (~2 μL) of the suspension was mixed with 10 μL of 10 mM KCl-HCl at pH 2.0 or 7.2 and dropped onto a coverslip for fluorescence microscope observation using a Nikon 90i fluorescence microscope (Tokyo, Japan). Scanning electron microscopic (SEM) observation of the microparticles was performed using a Hitachi S3500 microscope (Tokyo, Japan).

### pH-dependent insulin release from the encapsulated (coated) and nonencapsulated microparticles

For a typical release experiment, insulin or insulin-chitosan capsules coated by seven chitosan/heparin layer films (with chitosan as the outermost layer) were prepared, and particles without encapsulation were used as a control. Nanolayer-encapsulated capsules or bare particles were aliquoted (0.01 mL) into 0.3 mL pH 2, 3, 4, 5, 6, and 7.2 KCl/HCl buffer (10 mM; KCl was added to stabilize pH at strong acidic conditions) while agitating at room temperature (21°C), and after 2 hours, the capsules/particles were isolated by centrifugation (5,600× *g*, 3 minutes). Insulin release at different pH values before and after encapsulation was examined by measuring absorbance change in the supernatant at λ=280 nm with a UV-visible spectrophotometer, as described earlier.

### Animal studies

All animal procedures were approved by the King’s College London ethics committee and carried out under license in line with the UK Home Office Animals (Scientific Procedures) Act 1986.

The in vivo efficacy study of the insulin-loaded microcapsules was performed in a diabetic mouse model via oral gavage of 0.25 mL particle suspension in phosphate buffer (pH 5.5), using a 20 gauge feeding tube. ICR mice (25–30 g; Harlan Laboratories, Oxfordshire, UK), were made diabetic via a single intraperitoneal (ip) streptozotocin injection (180 mg/kg; in 10 mM citrate buffer at pH 4.5) 5–6 days before the tests of oral insulin formulations; only those animals with a nonfasting blood glucose concentration of 20 mmol/L or higher were used in the study. Unless specified, diabetic mice were fasted for 2 hours and remained fasted during the experiment, with free access to water. Experiments were also done with nonfasted animals. Blood samples were collected from the tail veins of mice before administering the drug and at different time intervals after dosing for measurement of the blood glucose concentration (Accu-Chek Aviva; Roche Diagnostics, Mannheim, Germany). The relative pharmacological activity of insulin after oral administration was calculated by comparing the area under the curve of the glucose level profile of the experiment group with that of direct ip administration, as recommended by Teply et al.^18^

We assessed the pharmacodynamic properties of the oral insulin formulations by an ip glucose tolerance test, using fasted (overnight) diabetic animals. Weight-matched, nondiabetic male ICR mice were used as the controls. Glucose concentrations were measured before an ip injection of 2 g/kg of glucose dissolved in saline solution and then after 0, 15, 30, 60, 90, 120, and 150 minutes after glucose injections. Dosages of oral insulin (250 μL; 30 and 60 units/kg for the fasted animals and 60 and 120 units/kg for the nonfasted animals) were given via a gavage needle 30 minutes before glucose ip injection.

Insulin concentrations were determined by a radioimmunoassay in plasma samples (10 μL) collected from the tail veins of mice, mixed with 100 μL 50 mM borate and ethylenediaminetetraacetic acid (EDTA) at pH 7.4 and centrifuged for 15 minutes at 2,000× *g*, using an in-house-generated antiserum against bovine insulin raised in a guinea pig (100% cross-reactivity to mouse insulin) and an ^125^I-labeled insulin in a homogeneous competitive format.^19^ Bovine ^125^I-insulin was prepared by the 1,3,4,6-tetrachloro-3α,6α-diphenylglucoluril method and purified by gel filtration. Purified rat insulin was used as the standard.^19^ Note that the immunoassay can only measure free insulin and part of bound insulin in plasma.

The following formulations were administered individually to the diabetic mice with doses of 30 and 60 units/kg (fasting) and 60 and 120 units/kg (fed): multilayer-coated capsule containing insulin microparticles, multilayer-coated capsule containing insulin-chitosan particles, insulin-alone microparticles, insulin-chitosan microparticles, and ip injection of the free-form insulin solution (20 IU/kg).

### Statistical analysis

Differences between groups were compared by a two-way analysis of variance (for curves) or student *t*-test (for means). Differences were considered to be statistically significant when the *P*-values were less than 0.05. Results are shown as mean ± standard deviation unless otherwise indicated.

## Results

### Optimization of insulin loading in colloidal microparticles

**Figure 2:**
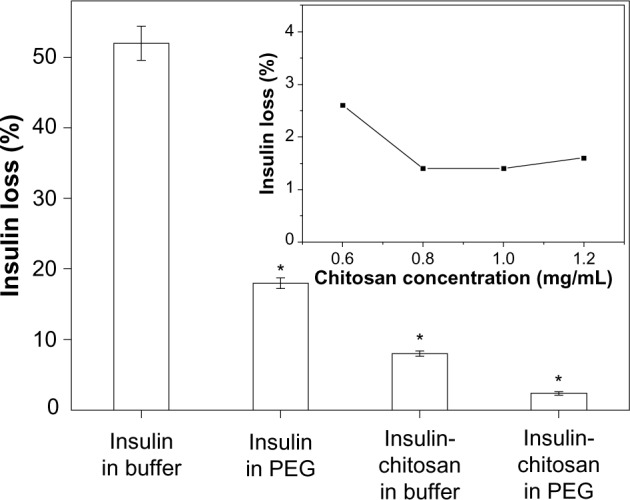
Insulin loss during the formation of microparticles by precipitation at pH 5.5. **Notes:** Most insulin was associated with the microparticles (least loss into solution) when precipitated with both chitosan and PEG (n=3; *P<0.001 versus insulin-alone particles in buffer). Inset: insulin loss as a function of the chitosan concentration in the precipitating solution. The ratio of volume of insulin and chitosan was kept constant at 1:1. **Abbreviation:** PEG, poly(ethylene) glycol.

In preliminary experiments, we established that insulin microparticles could be formed either by precipitation in the presence of nonionic polymer (PEG) or by complexes of insulin with chitosan. By combining PEG and chitosan precipitants, we achieved an enhanced and synergistic effect for producing insulin-containing precipitates. Figure 2 shows that more than 80% of insulin could be precipitated in the presence of 10% PEG 6000 compared with only about 50% in aqueous solutions. However, by the addition of chitosan (optimal concentration, 1 mg/mL; Figure 2 inset) into the PEG solution, a 98.5% insulin-association efficiency could be achieved with an insulin content (also called insulin loading capacity) of 90.9% (based on the ratio of insulin and chitosan used for forming complexes) in the insulin-chitosan microparticles.

**Figure 3:**
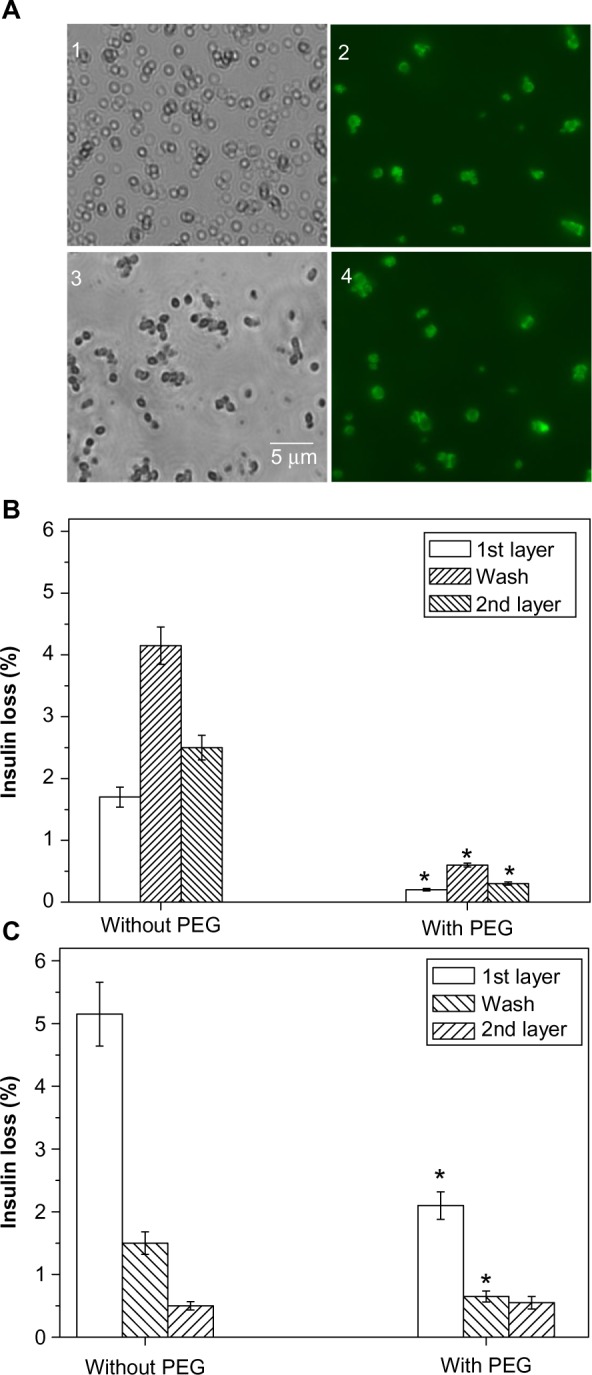
Insulin particle appearance and insulin loss during deposition of nanolayers. **Notes:** (**A**) Bright-field and fluorescence light microscope images of (1) the spherical insulin particles, (2) insulin particles coated with the first layer of chitosan-FITC, (3) insulin-chitosan particles, and (4) insulin-chitosan particles coated with the first layer of chitosan-FITC. The particles and the coating were prepared in the presence of 10% PEG 6000 (the final concentration after mixing). Note that images are from different batches of the particles. (**B** and **C**) Insulin loss during the deposition of the first two layers and the wash step. (**B**) Insulin, (**C**) insulin-chitosan (n=3; *P<0.001) in the presence of PEG versus without PEG. **Abbreviations:** PEG, poly(ethylene) glycol; FITC, fluorescein-5-isothiocyanate.

The mean diameter of the microparticles was ~1 μm. There was no notable difference in the particle size of insulin-alone and insulin-chitosan microparticles (Figure 3A, panels 1 and 3), although the insulin microparticles were loosely formed, whereas the insulin-chitosan particles were more solid and crystal-like in microscopic appearance.

### Deposition of multiple polysaccharide nanolayers on insulin microparticles

**Figure 4:**
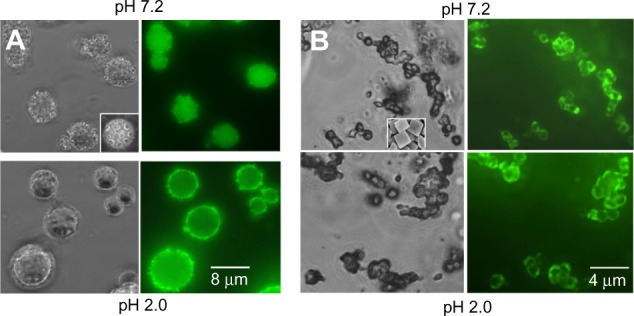
Bright-field and fluorescent images of the nanoencapsulated insulin (**A**) and nanoencapsulated insulin-chitosan (**B**) microparticles at pH 2.0 and 7.2, respectively. **Notes:** Left: bright-field; right: fluorescence. Insets: images of the microcrystals/microparticles, imaged using scanning electron microscopy.

Chitosan (pKa approximately 6.5, being positively charged at pH 5.5) was first deposited onto the insulin particles (isoelectric point of 5.4). Heparin (with negative charge) was subsequently deposited on the chitosan-layered surfaces via both electrostatic attraction and complexation. Figure 3A (panels 2 and 4) shows that the particles were coated individually with relatively uniform size and shape after the deposition of the nanolayers (FITC-labeled chitosan was used as the first layer to render the shell fluorescent). The insulin-alone microparticles, however, tended to aggregate together with each deposition step, and each capsule therefore usually contained several insulin microparticles. The light micrograph and SEM appearance of the insulin-alone and the insulin-chitosan particles with seven layers are shown in Figure 4A and B, where the crystalloid nature of the latter can be clearly seen (SEM images in insets).

### Reducing insulin loss during multilayer deposition

To avoid insulin particles being partially dissolved in the layer deposition solutions, PEG 6000 (10%) was added in the polysaccharide coating solutions to promote retention of the precipitated insulin, and therefore increase the capsule loading capacity. Figure 3B illustrates that addition of PEG reduced insulin loss during layer deposition: Without PEG, 1.6% of insulin was lost in the first chitosan deposition step, 4% in the first washing step, and 2.5% in the first heparin deposition step, giving a combined loss of 8.1%. In the presence of PEG, a combined loss of less than 1% was achieved after all three steps.

In the case of the insulin-chitosan microparticles (Figure 3C), 5% insulin was lost during the deposition of the first layer, 1.5% in the first washing step, and 0.5% in the first heparin step, giving a combined loss of 7% when no PEG was used. In the presence of PEG, the combined loss was approximately 3% after all three steps. The insulin encapsulation efficiency was 93.5% after the deposition of seven layers. Encapsulation efficiency is defined as the fraction of initial insulin encased by the capsules.

### Morphological changes of nanocoated insulin and insulin-chitosan particles in acidic and neutral conditions

Insulin and insulin-chitosan particles coated with seven layers of chitosan/heparin, with FITC-labeled chitosan as the first layer, were examined after exposure to pH 2.0 and 7.2 buffer solutions (Figure 4). The green fluorescence observed around the individual particles showed they were indeed coated with the chitosan and heparin. When the insulin capsules (Figure 4A) were exposed to a pH 7.2 buffer, the microparticles remained intact, and the morphology of the microparticles/capsules was not changed, indicating low solubility of the microparticles. At pH 2.0, however, the insulin capsules became round, likely because of the particle dissolution within the capsules, which also indicates substantial stability of the chitosan/heparin coating layers at the acidic conditions (ability to retain the dissolved insulin). We found that a coating with less than six layers was unstable at pH 2.0, probably because of incomplete coverage of the particles.

For the encapsulated insulin-chitosan particles (Figure 4B), the particles remained intact, and there was no difference in crystalloid morphology observed at both pH 7.2 and 2.0, indicating that the solubility of the insulin-chitosan complexes was little affected under the acidic pH conditions.

### In vitro insulin release over pH range 2.0–7.2

**Figure 5:**
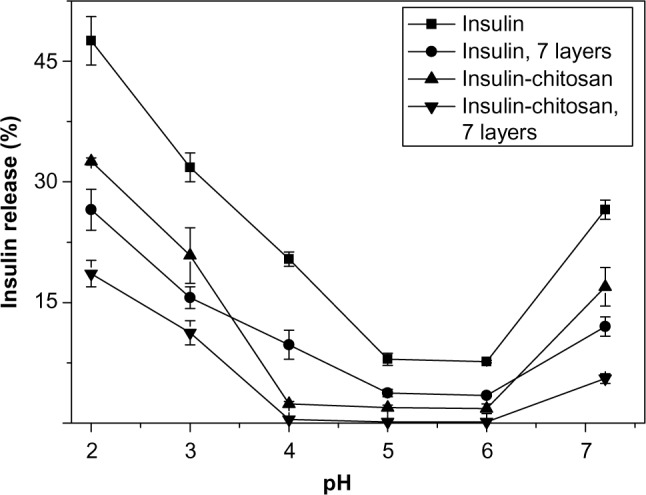
pH-sensitive insulin release in vitro from the insulin and insulin-chitosan particles with and without nanoencapsulation with seven layers (n=3). **Notes:** Release time studied was 2 hours. All data points *P*<0.001 versus insulin-alone particles. *P*-levels are not shown on the graph for clarity.

Because insulin dissolved in solutions is vulnerable to digestive enzymatic digestion, we did not use the simulated gastric fluid or simulated intestinal fluid for the release experiments. Figure 5 shows insulin release in vitro from the insulin-alone particles and the insulin-chitosan microparticles at different pH conditions before and after multilayer encapsulation. Insulin release was minimal in the pH range of 5–6, which is close to the pH of insulin. The solubility of the particles increased substantially at both acidic pH and at pH greater than 7. The lowest insulin release was seen with a combination of insulin-chitosan complexation and nanocoating at both extremes of pH. Insulin alone without nanolayers had the highest release rate.

At pH 2.0, there was a marked decrease of solubility by 32% when the insulin-chitosan complexation was compared with the insulin-alone particles. Furthermore, a seven-layer chitosan-heparin nanofilm coating resulted in a further 42% decrease in solubility for both types of microparticles.

### In vivo study of the effects of orally administered encapsulated insulin and insulin-chitosan particles in diabetic mice

**Figure 6:**
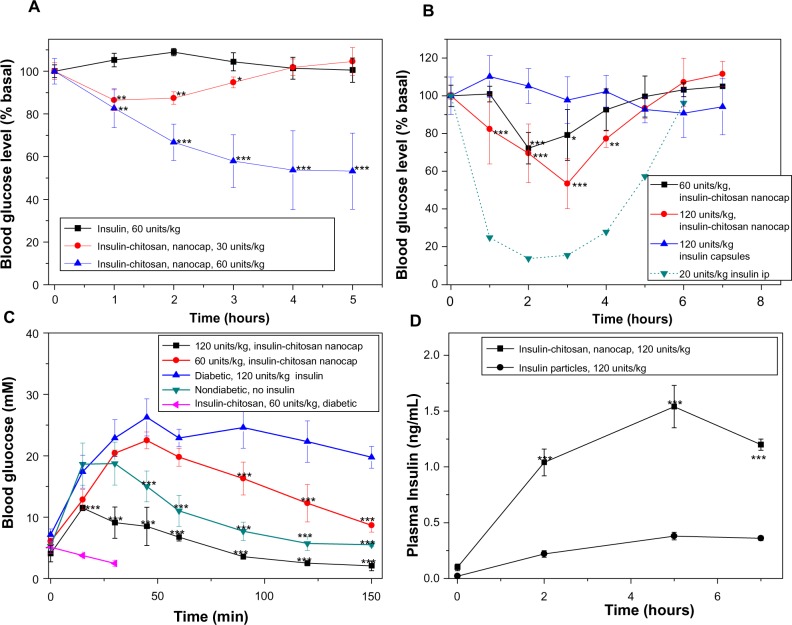
Blood glucose level changes relative to the baseline values after the oral insulin and insulin-chitosan microparticles (encapsulated with seven layers and controls) in diabetic mice. **Notes:** (**A**) Fasted mice, (**B**) fed mice, and (**C**) effect of the oral insulin and insulin-chitosan on the blood glucose levels after intraperitoneal glucose challenge in the overnight-fasted diabetic and nondiabetic mice (n=4 or 5). (**D**) Plasma insulin level after the oral administration of insulin (control) and insulin-chitosan particles (nanoencapsulated with seven layers; n=4). *P<0.05; **P<0.01; ***P<0.001, insulin-chitosan versus insulin-alone capsules. **Abbreviations:** ip, intraperitoneal; min, minutes; nanocap, nanoencapsulated.

With fasted diabetic mice, oral administration of 60 units/kg of the encapsulated insulin-chitosan formulation produced a maximum of 50% reduction of the blood glucose level, which lasted for at least 5 hours (Figure 6A). An oral dose of 30 units/kg of the encapsulated insulin-chitosan resulted in an approximately 15% decrease of blood glucose levels, which lasted about 2–3 hours (Figure 6A). Oral insulin alone had no hypoglycemic effect.

With fed mice, a higher oral dose of the encapsulated insulin-chitosan was required to lower blood glucose levels (Figure 6B): A 60 units/kg dose lowered mean blood glucose levels by a maximal 30% 2 hours after administration of insulin, lasting about 4 hours, and a 120 units/kg dose provided a maximal 50% decrease of blood glucose at 3 hours after administration of insulin, with the hypoglycemic effect lasting about 5 hours. The relative pharmacological activity of insulin of the encapsulated insulin-chitosan was 6.9% for a dose of 120 units/kg.

When the encapsulated oral insulin-chitosan formulations were given to overnight-fasted diabetic mice (blood glucose decreased to below 7 mM) receiving an ip glucose load, the best control of glycemia was seen with a dose of 120 units/kg (Figure 6C). For diabetic mice receiving an oral dose of 60 units/kg of the nonencapsulated insulin-chitosan microparticles, blood glucose did not increase after the glucose challenge but continued to decrease to below 3 mM, indicating a faster onset of action. The experiment was then stopped because of the severe hypoglycemic effect on the animals.

The intestinal absorption of insulin from the orally administered encapsulated (seven layers) insulin and insulin-chitosan (120 units/kg) was also evaluated by measuring plasma insulin levels in the fasted diabetic mice (Figure 6D). Mice receiving insulin-alone capsules showed only a slight increase in plasma insulin concentration postadministration. However, after oral administration of the insulin-chitosan capsules, the plasma insulin levels had increased markedly at 2, 5, and 6 hours (*P*<0.001 versus insulin alone), with an insulin peak (C_max_=1.53 ng/mL) at 5 hours postadministration compared with 0.37 ng/mL for the insulin-alone capsules (*P*<0.001).

## Discussion

In this study, we report a method for improving both the in vitro release behavior and the in vivo safety and efficacy of the orally administered insulin, using multilayered encapsulation of a precipitated chitosan-insulin complex.

The average size of the insulin-chitosan particles formed in the presence of 10% PEG was ∼1 μm. Although further reduction of the particle size to submicron levels might be possible by using emulsification and homogenization protocols,^20^ polyelectrolyte-induced bridging between particles may cause problems of particle aggregation after multiple steps of centrifugation.^21^ We therefore considered that a smaller particle size was not a desirable strategy for the purpose of this study.

LBL deposition generates an organized nanothin film or coating from the application of alternating oppositely charged (usually polymer) species and is an appealing means of preparing robust containments (capsules) of high stability and tunable permeability.^22^,^23^ The coating layers can be fabricated with a thickness in the order of nanometers, enabling the design and engineering of surfaces and interfaces at the molecular level. Originally developed as a coating technology for flat surfaces, LBL technology has been now extensively used for encapsulation of particles, proteins, and cells.^24^

Using drug crystals or nanoparticle aggregates as core templates for encapsulation is an established application of the LBL nanocoating to control the release of drugs from micro- or nanoparticles.^25^,^26^ For the nanofilms in the present study, we used the anionic polymer, heparin, with a strongly ionic sulphate group in complexation with amine groups of chitosan; this pairing is known to contribute greatly to the stability of the layered nanofilm,^27^ making it a very stable coating even under harsh gastric (acidic) conditions. The improved stability of the multilayer coating was confirmed in this study in vitro, where the insulin-based chitosan/heparin (seven layers) microcapsules remained intact at pH 2.0, even after the insulin microparticles were dissolved. This substantial stability of the nanocoating layers may be important for more predictable release of insulin (with the absence of unpredictable hypoglycemia) after oral administration than was the case with the insulin-chitosan alone, which we demonstrated in this study (Figure 5). Of note, we found that a nanocoating consisting of less than six layers was not stable enough to retain insulin content in the hollow capsule at pH 2.0. The mean thickness of LBL nanofilms of seven layers for encapsulation was estimated to be ~50 nm, according to our previous investigation.^28^ Note that this estimation was made on the basis that the substrate has usually little effect on the thickness of the multilayers formed by LBL technique.

We also observed that addition of PEG at pH 5.5 to the precipitating and coating solutions substantially improved the loading (90.9%, see earlier) and encapsulation efficiency (93.5%) and minimized the insulin loss during the capsule preparation. PEG has been previously used to prevent interactions and therefore reduce solubility in the LBL nanoencapsulation of highly soluble proteins.^29^–^31^ Insulin is highly soluble at high and low pH conditions but is poorly dissolved at pH 5.5 (near the pH of insulin). The solubility was further decreased in our study by the formation of a chitosan-insulin complex with PEG in the precipitation medium. Moreover, by avoiding the use of coprecipitants that have been used by previous investigators, such as tripolyphosphate,^32^,^33^ poly-γ-glutamic acid,^8^ and poloxamar,^10^ as well as organic solvents, and with minimized use of chitosan (the concentration of which was 1 mg/mL compared with insulin, with a concentration of 10 mg/mL) in the formation of microprecipitates, we were able to improve the loading capacity of insulin in the microparticles to more than 90%. PEG is known to have good biocompatibility and minimal toxicity and is approved by the US Food and Drug Administration for human use.^34^ Chitosan is a natural biopolymer that is nonimmunogenic, nontoxic, and biodegradable and has already seen wide application in drug delivery.^35^

Insulin-chitosan microparticles without coating layers have previously been reported to be without toxicological effects^36^ and to be effective in lowering blood glucose after oral administration to diabetic animals. However, the onset of action was much faster and more intense compared with the encapsulated particles here, leading to high risk of severe hypoglycemia in the animals. This is likely because of the poor acid stability of the unencapsulated insulin-chitosan microparticles, leading to uncontrollable burst release and subsequent acute biological responses. Multilayer nanocoatings of chitosan/albumin^10^ or Fe^3+^/dextran sulphate^11^ have been previously used as the dissolution barrier/sacrificial layer, allowing control over the release kinetics of insulin from microcarriers. However, these coating structures may have poor structural stability at acidic pH conditions as a result of loss of net negative charges in the ionic binding pairs. In contrast, the chitosan/heparin multilayer nanofilms we used to coat the insulin-chitosan microparticles were shown clearly in Figure 6C to have an initial release and action that was delayed, promising greater clinical safety because of the more controlled absorption rate and subsequently lower risk of problematic hypoglycemia after oral administration.

Our study has some limitations. First, although we showed a dose-related effect of the encapsulated oral insulin formulation at 60 and 120 units/kg, we have not yet studied the dose-response relationship in detail. Second, although we showed good effect of chitosan in the capsules and in complexation with insulin, we have not yet systematically optimized oral insulin delivery by researching chemical modifications of chitosan that may enhance its mucoadhesive and/or tight junction-opening properties (eg, trimethylated and thiolated chitosan derivatives^37^), which may be the subject of our further studies. Improved intestinal permeation afforded by such chitosans may increase the pharmacological activity, which was low in our study, although comparable with previously reported oral insulin formulations.^37^ Our encapsulated oral insulin also now needs to be tested for its pharmacological and toxicological properties and hypoglycemic efficacy in larger animals before proceeding to the first clinical studies.

## Conclusion

Chitosan/heparin nanolayer encapsulation was found to greatly enhance the acid stability of insulin-incorporated chitosan microparticles and improve insulin release behavior, effectively lowering blood glucose concentrations in diabetic mice. Nanolayer encapsulation is novel. It helped reduce the possibility of rapid fall of blood glucose levels, making the formulation safer for oral administration. Meanwhile, the addition of PEG in the precipitating and coating solutions improved insulin loading and minimized undesirable loss of the protein resulting from redissolution. This technique represents a promising strategy to promote the intestinal absorption efficiency of the hormone, enabling an efficient route for oral delivery of insulin for diabetes management. More extensive testing in clinically relevant animal models is now needed before progressing to clinical development.
